# Correction: Changes in pneumococcal vaccine coverage in the Canadian Longitudinal Study on Aging (CLSA): An analysis based on the 2018–2021 follow-up 2 survey

**DOI:** 10.1371/journal.pone.0343252

**Published:** 2026-02-17

**Authors:** Giorgia Sulis, Nawal Maredia, Christina Wolfson, Nicole E. Basta

There is an error in Table 2, the column heading for Individuals aged 65 and older (N = 3733) is incorrectly labeled as Newly Unvaccinated. The correct label should be Newly Vaccinated. Please see the correct Table 2 here.

**Table 2:** Characteristics of Canadian Longitudinal Study on Aging (CLSA) participants who reported being newly vaccinated for pneumococcal disease at follow-up 2 (FUP2) since FUP1, versus those still non-vaccinated. Only participants who were considered vaccine-eligible at both FUP1 and FUP2 and who reported not beig vaccinated at FUP1 were included in this analysis.

Characteristic	Self-reported pneumococcal vaccination status in lifetime							
	Individuals aged 65 and older (N = 3733)				Individuals aged <65 with at least 1 CMC (N = 2776)			
	Still Unvaccinated		Newly Vaccinated		Still Unvaccinated		Newly Vaccinated	
	N	% (95% CI)	N	% (95% CI)	N	% (95% CI)	N	% (95% CI)
Overall	2672	71.6 (70.1-73.0)	1061	28.42 (27.0-29.9)	2470	89.0 (87.8-90.1)	306	11.0 (9.9-12.2)
Sex at birth								
Female	1218	69.5 (67.3-71.6)	535	30.5 (28.4-32.7)	1285	89.2 (87.5-90.7)	156	10.8 (9.3-12.5)
Male	1454	73.4 (71.4-75.3)	526	26.6 (24.7-28.6)	1185	88.8 (87.0-90.4)	150	11.2 (9.6-13.0)
Age group								
< 55	N/A	N/A	N/A	N/A	398	91.3 (88.2-93.6)	38	8.7 (6.4-11.8)
55-64	N/A	N/A	N/A	N/A	2072	88.5 (87.2-89.8)	268	11.5 (10.2-12.8)
65-74	1419	68.8 (66.7-70.7)	645	31.3 (29.3-33.3)	N/A	N/A	N/A	N/A
75-84	978	74.5 (72.1-76.8)	334	25.5 (23.2-27.9)	N/A	N/A	N/A	N/A
85+	275	77.0 (72.4-81.1)	82	23.0 (18.9-27.6)	N/A	N/A	N/A	N/A
Racialized								
No	2534	71.5 (70.0-72.9)	1011	28.5 (27.1-30.0)	2295	89.0 (87.8-90.2)	283	11.0 (9.8-12.2)
Yes	134	73.2 (66.3-79.1)	49	26.8 (20.9-33.7)	175	88.8 (83.6-92.5)	22	11.2 (7.5-16.4)
Missing	4	80.0 (30.9-97.3)	1	20.0 (2.7-69.1)	0	0.0 (N/A)	1	100.0 (N/A)
Highest education level								
Less than second. school educ.	191	76.1 (70.4-81.0)	60	23.9 (19.0-29.6)	57	81.4 (70.6-88.9)	13	18.6 (11.1-29.4)
Second. school grad., no post-second. school educ.	251	71.5 (66.6-76.0)	100	28.5 (24.0-33.4)	179	86.9 (81.6-90.9)	27	13.1 (9.1-18.4)
Some post-second. educ.	230	74.9 (69.8-79.5)	77	25.1 (20.5-30.2)	178	90.4 (85.4-93.8)	19	9.6 (6.2-14.6)
Post-second. degree/diploma	1989	70.8 (69.0-72.4)	822	29.2 (27.6-31.0)	2056	89.3 (87.9-90.5)	247	10.7 (9.5-12.1)
Missing	11	84.6 (54.9-96.1)	2	15.4 (3.9-45.1)	0	N/A	0	N/A
Annual household income (in Canadian dollars)								
Less than $20,000	162	76.8 (70.6-82.0)	49	23.2 (18.0-29.4)	88	88.9 (81.0-93.7)	11	11.1 (6.3-19.0)
$20,000 to <$50,000	783	77.4 (74.8-79.9)	228	22.6 (20.1-25.2)	282	87.6 (83.5-90.8)	40	12.4 (9.2-16.5)
$50,000 to <$100,000	937	68.1 (65.6-70.6)	438	31.9 (29.4-34.4)	702	88.5 (86.1-90.6)	91	11.5 (9.4-13.9)
$100,000 to <$150,000	357	67.5 (63.4-71.3)	172	32.5 (28.7-36.6)	591	89.5 (87.0-91.7)	69	10.5 (8.3-13.0)
$150,000 or higher	181	68.0 (62.2-73.4)	85	32.0 (26.6-37.8)	685	90.6 (88.3-92.5)	71	9.4 (7.5-11.7)
Missing	252	73.9 (69.0-78.3)	89	26.1 (21.7-31.0)	122	83.6 (76.6-88.7)	24	16.4 (11.3-23.4)
Marital/partner status								
Single/never married/never lived with a partner	187	70.6 (64.8-75.7)	78	29.4 (24.3-35.2)	269	87.3 (83.1-90.6)	39	12.7 (9.4-16.9)
Married/Common-law	1613	70.2 (68.3-72.0)	686	29.8 (28.0-31.7)	1786	88.8 (87.4-90.1)	225	11.2 (9.9-12.6)
Widowed	457	74.8 (71.2-78.1)	154	25.2 (21.9-28.8)	65	90.3 (81.0-95.3)	7	9.7 (4.7-19.0)
Divorced/Separated	413	74.3 (70.5-77.7)	143	25.7 (22.3-29.5)	348	90.9 (87.5-93.4)	35	9.1 (6.6-12.5)
Missing	2	100.0 (N/A)	0	0.0 (N/A)	2	100.0 (N/A)	0	0.0 (N.A)
Province of residence								
Newfoundland	304	78.4 (74.0-82.2)	84	21.6 (17.8-26.0)	210	94.6 (90.7-96.9)	12	5.4 (3.1-9.3)
Nova Scotia	276	76.5 (71.8-80.5)	85	23.5 (19.5-28.2)	169	88.5 (83.1-92.3)	22	11.5 (7.7-16.9)
Quebec	543	73.6 (70.3-76.6)	195	26.4 (23.4-29.7)	508	89.0 (86.1-91.3)	63	11.0 (8.7-13.9)
Ontario	543	67.0 (63.7-70.2)	267	33.0 (29.8-36.3)	546	86.3 (83.3-88.7)	87	13.7 (11.3-16.7)
Manitoba	204	71.3 (65.8-76.3)	82	28.7 (23.7-34.2)	228	93.4 (89.6-95.9)	16	6.6 (4.1-10.4)
Alberta	200	65.1 (59.6-70.3)	107	34.9 (29.7-40.4)	251	86.6 (82.1-90.0)	39	13.4 (10.0-17.9)
British Columbia	602	71.4 (68.3-73.0)	241	28.4 (27.0-29.9)	558	89.3 (86.6-91.5)	67	10.7 (8.5-13.4)
Urbanicity of residence								
Urban	2438	71.3 (69.8-72.8)	981	28.7 (27.2-30.2)	2232	88.7 (87.5-89.9)	283	11.3 (10.1-12.5)
Rural	224	74.4 (69.2-79.0)	77	25.6 (21.0-30.8)	230	91.6 (87.5-94.5)	21	8.4 (5.5-12.5)
Missing	10	76.9 (47.8-92.4)	3	23.1 (7.6-52.2)	8	80.0 (45.9-95.0)	2	20.0 (5.0-54.1)
Chronic Medical Condition status								
None reported	704	74.3 (71.5-77.0)	243	25.7 (23.0-28.5)	236	93.3 (89.5-95.8)	17	6.7 (4.2-10.5)
At least one reported	1951	70.6 (68.9-72.3)	811	29.4 (27.7-31.1)	2470	89.0 (87.8-90.1)	306	11.0 (9.9-12.2)
Missing	17	70.8 (50.2-85.4)	7	29.2 (14.6-49.8)	20	95.2 (72.8-99.3)	1	4.8 (0.7-27.2)
Contact with family doctor in previous 12 months								
No	293	85.2 (81.0-88.6)	51	14.8 (11.4-19.0)	267	94.7 (91.4-96.8)	15	5.3 (3.2-8.6)
Yes	2374	70.2 (68.6-71.7)	1009	29.8 (28.3-31.4)	2200	88.3 (87.0-89.5)	291	11.7 (10.5-13.0)
Missing	5	83.3 (36.9-97.7)	1	16.7 (2.3-63.1)	3	100.0 (N/A)	0	0.0 (N/A)
Receipt of influenza vaccine in previous 12 months (self-reported)								
No	1355	88.9 (87.2-90.3)	170	11.1 (9.7-12.8)	1295	94.8 (93.5-95.9)	71	5.2 (4.1-6.5)
Yes	1313	59.7 (57.6-61.7)	888	40.3 (38.3-42.4)	1172	83.4 (81.3-85.2)	234	16.6 (14.8-18.7)
Missing	4	57.1 (23.0-85.6)	3	42.9 (14.4-77.0)	3	75.0 (23.8-96.7)	1	25.0 (3.3-76.2)
