# Supplementary material for: Changes in pneumococcal vaccine coverage in the Canadian Longitudinal Study on Aging (CLSA): An analysis based on the 2018–2021 follow-up 2 survey
Source: PLoS One. 2026 Jan 23;21(1):e0338213. doi: 10.1371/journal.pone.0338213 (PMC12829781; doi:10.1371/journal.pone.0338213)
Supplement: S1 Table — For each variable, we report the corresponding survey question and Canadian Longitudinal Study on Aging (CLSA) variable name, response options offered to participants during the survey, and categorization used for the purpose of this study. (PDF) [file pone.0338213.s001.pdf]

S1 Table: Description of study variables. For each variable, we report the corresponding survey question and Canadian Longitudinal Study on Aging (CLSA) variable name, response options offered to participants during the survey, and categorization used for the purpose of this study.

Variable	Survey question [CLSA variable name]	Possible responses	Categorization for analysis
**OUTCOMES**			
Self-reported pneumococcal vaccination as of FUP2	Have you had a pneumonia shot (pneumococcal vaccination) in your life? [PHB_PCV_COF2]	1-Yes	Vaccinated
		2-No	Unvaccinated
		Don't know/No answer/Refused/Missing	Missing
Newly vaccinated for pneumococcal disease at FUP2 relative to FUP1	Variable derived from:<br><br>Have you had a pneumonia shot (pneumococcal vaccination) in your life? [PHB_PCV_COF2]<br><br>And<br><br>Have you had a pneumonia shot (pneumococcal vaccination) in your life? [PHB_PCV_COF1]	As above	Yes – newly vaccinated (if PHB_PCV_COF2="yes" and PHB_PCV_COF1="no")
			No – still unvaccinated (if PHB_PCV_COF2="no" and PHB_PCV_COF1="no")
			Missing (if PHB_PCV_COF2 and/or PHB_PCV_COF1 missing)
**SOCIODEMOGRAPHIC CHARACTERISTICS**			
Sex at birth	Are you male or female? [SEX_ASK_COM]	Female	Female
		Male	Male
Age group at FUP2	Age (years) – Calculated: Date of interview less reported Date of Birth [AGE_NMBR_COF2]	Continuous number	<50
			50-54
			55-64
			65-74
			75-84
			85+
Age group at FUP1	Age (years) – Calculated: Date of interview less reported Date of Birth [AGE_NMBR_COF1]	Continuous number	<50
			50-54
			55-64
			65-74

Variable	Survey question [CLSA variable name]	Possible responses	Categorization for analysis
			75-84
			85+
Racialized	Cultural/racial background [SDC_DCGT_COM]	1-White only	Yes
		2-Black only	No
		3-Korean only	
		4-Filipino only	
		5-Japanese only	
		6-Chinese only	
		7-South Asian only	
		8-Southeast Asian only	
		9-Arab only	
		10-West Asian only	
		11-Latin American only	
		12-Other racial or cultural original (only)	
		13-Multiple racial or cultural origins	
		Missing	Missing
Highest education level	Highest level of education [ED_UDR04_COM]	1-Less than secondary school graduation	Less than secondary school graduation
		2-Secondary school graduation, no post-secondary education	Secondary school graduation, no post-secondary education
		3-Some post-secondary education	Some post-secondary education
		4-Post-secondary degree/diploma	Post-secondary degree/diploma
		Missing	Missing
Annual household income	What is your best estimate of the total household income received by all household members, from all sources, before taxes and deductions, in the past 12 months? [INC_TOT_COF2]	1-Less than $20,000	<$20,000
		2-$20,000 or more, but less than $50,000	$20,000 to <$50,000
		3-$50,000 or more, but less than $100,000	$50,000 to <$100,000
		4-$100,000 or more, but less than $150,000	$100,000 to <$150,000
		5-$150,000 or more	$150,000 or higher
		Don’t know/No answer/Refused/Missing	Missing
Marital/partner status	What is your current marital/partner status? [SDC_MRTLF2_COF2]	1-Single, never married or never lived with a partner	Single/Never married/Never lived with a partner
		2-Married, living with a partner in a common-law relationship	Married/Common-law
		3-Widowed	Widowed

Variable	Survey question [CLSA variable name]	Possible responses	Categorization for analysis
		4-Divorced	Divorced/Separated
		5-Separated	
		Don't know/No answer/Refused/Missing	Missing
Province of residence	Province of residence [WGHTS_PROV_COF2]	10-Newfoundland	Newfoundland
		11-Prince Edward Island	Prince Edward Island
		12-Nova Scotia	Nova Scotia
		13-New Brunswick	New Brunswick
		24-Quebec	Quebec
		35-Ontario	Ontario
		46-Manitoba	Manitoba
		47-Saskatchewan	Saskatchewan
		48-Alberta	Alberta
		59-British Columbia	British Columbia
Urbanicity of residence	Urban/rural classification [SDC_URBAN_RURAL_COF2]	0-Rural area	Rural
		9-Postal code link to Dissemination Area	
		1-Urban core	Urban
		2-Urban fringe	
		4-Urban areas out CMAs/CAs	
		6-Secondary urban core	
		Missing	Missing
**VARIABLES RELATED TO HEALTH STATUS AND HEALTHCARE UTILIZATION**			
Self-reported- at least one chronic medical condition (assessed at both FUP1 and FUP2)	Cardiovascular disease:<br><br>Has a doctor ever told you that you have…<br>- heart disease (including congestive heart failure or CHF)? [CCC_HEART_COF1, CCC_HEART_COF2]	Yes/No/Missing	Yes if "yes" to at least one of the relevant questions<br><br>No if "no" to all relevant questions<br><br>Missing if all "missing"
	- heart attack or myocardial infarction? [CCC_AMI_COF1, CCC_AMI_COF2]	Yes/No/Missing	

Variable	Survey question [CLSA variable name]	Possible responses	Categorization for analysis
	- angina (or chest pain due to heart disease)? [CCC_ANGI_COF1, CCC_ANGI_COF2]	Yes/No/Missing	
	- high blood pressure or hypertension? [CCC_HBP_COF1, CCC_HBP_COF2]	Yes/No/Missing	
	Chronic lung disease:<br><br>Has a doctor ever told you that you have:<br>- any of the following: emphysema, chronic bronchitis, chronic obstructive pulmonary disease (COPD), or chronic changes in lungs due to smoking? [CCC_COPD_COF1, CCC_COPD_COF2]	Yes/No/Missing	
	- asthma? [CCC_ASTHM_COF1, CCC_ASTHM_COF2]	Yes/No/Missing	
	Cerebrovascular disease:<br><br>Has a doctor ever told you that you have experienced:<br>- a stroke or cerebrovascular accident (CVA)? [CCC_CVA_COF1, CCC_CVA_COF2]	Yes/No/Missing	
	- a ministroke or TIA (transient ischemic attack)? [CCC_TIA_COF1, CCC_TIA_COF2]	Yes/No/Missing	
	Chronic kidney disease:<br><br>Has a doctor ever told you that you have kidney disease or kidney failure? [CCC_KIDN_COF1, CCC_KIDN_COF2]	Yes/No/Missing	
	Diabetes mellitus:	Yes/No/Missing	

Variable	Survey question [CLSA variable name]	Possible responses	Categorization for analysis
	Has a doctor ever told you that you have diabetes, borderline diabetes or that your blood sugar is high? [DIA_DIAB_COF1, DIA_DIAB_COF2]		
	Cancer<br><br>Has a doctor ever told you that you had cancer? [CCC_CANC_COF1, CCC_CANC_COF2]	Yes/No/Missing	
	Chronic neurologic condition:<br><br>Has a doctor ever told you that you have…<br>- dementia or Alzheimer's disease? [CCC_ALZH_COF1, CCC_ALZH_COF2]	Yes/No/Missing	
	- Parkinson's disease? [CCC_PARK_COF1, CCC_PARK_COF2]	Yes/No/Missing	
	- multiple sclerosis? [CCC_MS_COF1, CCC_MS_COF2]	Yes/No/Missing	
Contact with a family doctor in previous 12 months (assessed at both FUP1 and FUP2)	During the past 12 months, have you had contact with a family doctor? [HCU_FAMPHY_COF1, HCU_FAMPHY_COF2]	1-Yes	Yes
		2-No	No
		Don't know/No answer/Refused/Missing	Missing
Self-reported influenza vaccination in previous 12 months (assessed at both FUP1 and FUP2)	Have you had a flu shot in the last 12 months? [PHB_FLUV_COF1, PHB_FLUV_COF2]	1-Yes	Yes
		2-No	No
		Don't know/No answer/Refused/Missing	Missing
