# Supplementary material for: Changes in pneumococcal vaccine coverage in the Canadian Longitudinal Study on Aging (CLSA): An analysis based on the 2018–2021 follow-up 2 survey
Source: PLoS One. 2026 Jan 23;21(1):e0338213. doi: 10.1371/journal.pone.0338213 (PMC12829781; doi:10.1371/journal.pone.0338213)
Supplement: S2 Table — (PDF) [file pone.0338213.s002.pdf]

S2 Table. Self-reported pneumococcal vaccination status (vaccinated or unvaccinated during lifetime) among Canadian Longitudinal Study on Aging (CLSA) comprehensive cohort participants who were considered eligible to receive a pneumococcal vaccine as per Canada's National Advisory Committee on Immunization (NACI) guidelines, by key sociodemographic characteristics at follow-up 1 (FUP1; 2015-2018).

Characteristic	Self-reported pneumococcal vaccination in lifetime							
	Individuals aged 65 and older (N=13,366)				Individuals aged < 65 with at least one CMC (N=6,714)			
	Vaccinated		Unvaccinated		Vaccinated		Unvaccinated	
	N	% (95% CI)	N	% (95% CI)	N	% (95% CI)	N	% (95% CI)
**Overall**	7180	53. 7 (52.9-54.6)	6186	46.3 (45.4-47.1)	1157	17.2 (16.3-18.2)	5557	82.8 (81.8-83.7)
**Sex at birth**								
Female	3856	56.7 (55.5-57.8)	2948	43.3 (42.2-44.5)	614	17.6 (16.4-18.9)	2869	82.4 (81.1-83.6)
Male	3324	50.7 (49.4-51.9)	3238	49.3 (48.1-50.6)	543	16.8 (15.6-18.1)	2688	83.2 (81.9-84.4)
Missing	0	0.0 (N/A)	0	0.0 (N/A)	0	0.0 (N/A)	0	0.0 (N/A)
**Age group**								
<55	N/A	N/A	N/A	N/A	202	11.7 (10.3-13.3)	1527	88.3 (86.7-89.7)
55-64	N/A	N/A	N/A	N/A	955	19.2 (18.1-20.3)	4030	80.8 (79.7-81.9)
65-74	3653	46.9 (45.8-48.0)	4141	53.1 (52.0-54.2)	N/A	N/A	N/A	N/A
75-84	2947	62.8 (61.4-64.2)	1747	37.2 (35.8-38.6)	N/A	N/A	N/A	N/A
85+	580	66.1 (62.9-69.1)	298	33.9 (30.9-37.1)	N/A	N/A	N/A	N/A
**Racialized**								
No	6919	54.1 (53.2-54.9)	5880	45.8 (45.1-46.8)	1087	17.4 (16.4-18.3)	5174	82.6 (81.7-83.6)
Yes	253	45.8 (41.6-49.9)	300	54.2 (50.1-58.4)	70	15.5 (12.4-19.1)	382	84.5 (80.9-87.6)
Missing	8	57.1 (31.6-79.4)	6	42.9 (20.6-68.4)	0	0.0 (N/A)	01	100 (N/A)
**Highest education level**								
Less than second. school educ.	537	55.1 (51.9-58.2)	438	44.9 (41.8-48.1)	39	19.6 (14.7-25.7)	160	80.4 (74.3-85.3)
Second. school grad., no post-second. school educ.	746	54.3 (51.7-56.9)	628	45.7 (43.1-48.3)	111	19.3 (16.3-22.8)	463	80.7 (77.2-83.7)
Some post-second. educ.	556	53.2 (50.2-56.2)	486	46.8 (43.8-49.8)	106	21.4 (18.0-25.2)	389	78.6 (74.8-82.0)
Post-second. degree/diploma	5323	53.6 (52.6-54.6)	4613	46.4 (45.4-47.4)	901	16.6 (15.6-17.6)	4543	83.4 (82.4-84.4)
Missing	18	50.0 (34.2-65.8)	18	50.0 (34.2-65.8)	0	0.0 (N/A)	02	100 (N/A)
**Annual household income (in Canadian dollars)**								
Less than $20,000	346	48.1 (44.5-51.8)	373	51.9 (48.2-55.5)	91	28.6 (23.9-33.8)	227	71.4 (66.2-76.1)

Characteristic	Self-reported pneumococcal vaccination in lifetime							
	Individuals aged 65 and older (N=13,366)				Individuals aged < 65 with at least one CMC (N=6,714)			
	Vaccinated		Unvaccinated		Vaccinated		Unvaccinated	
	N	% (95% CI)	N	% (95% CI)	N	% (95% CI)	N	% (95% CI)
$20,000 to <$50,000	1932	51.6 (50.0-53.2)	1809	48.4 (46.8-50.0)	166	19.0 (16.5-21.7)	708	81.0 (78.3-83.5)
$50,000 to <$100,000	2800	55.2 (53.8-56.5)	2274	44.8 (43.5-46.2)	378	18.2 (16.6-19.9)	1700	81.8 (80.1-83.4)
$100,000 to < $150,000	1009	55.7 (53.4-58.0)	801	44.3 (42.0-46.6)	222	14.6 (12.9-16.4)	1302	85.4 (83.6-87.1)
$150,000 or higher	498	52.0 (48.8-55.1)	460	48.0 (44.9-51.2)	241	14.7 (13.1-16.5)	1399	85.3 (83.5-86.9)
Missing	595	55.9 (52.9-58.9)	469	44.1 (41.1-47.1)	59	21.1 (16.7-26.2)	221	78.9 (73.8-83.3)
**Marital/partner status**								
Single/never married/never lived with a partner	453	50.8 (47.5-54.1)	439	49.2 (45.9-52.5)	180	21.5(18.9-24.4)	657	78.5 (75.6-81.1)
Married/Common-law	4468	53.1 (52.0-54.2)	3944	46.9 (45.8-48.0)	775	16.1 (15.1-17.2)	4024	83.9 (82.8-84.9)
Widowed	1405	61.2 (59.2-63.2)	889	38.8 (36.8-40.8)	44	23.8 (18.2-30.4)	141	76.2 (69.6-81.8)
Divorced/Separated	852	48.3 (46.0-50.6)	912	51.7 (49.4-54.0)	158	17.7 (15.4-20.4)	733	82.3 (79.6-84.6)
Missing	2	50.0 (12.3-87.7)	2	50.0 (12.3-87.7)	0	0.0 (N/A)	02	100 (N/A)
**Province of residence**								
Newfoundland	276	29.5 (26.7-32.5)	659	70.5 (67.5-73.3)	48	9.2 (7.0-11.9)	476	90.8 (88.1-93.0)
Nova Scotia	682	51.5 (48.9-54.2)	641	48.5 (45.8,51.1)	104	19 (16.0-22.6)	442	81 (77.4-84.0)
Quebec	1516	56.7 (54.8-58.5)	1160	43.3 (41.5-45.2)	232	16.8 (14.9-18.9)	1149	83.2 (81.1-85.1)
Ontario	1568	54.1 (52.3-55.9)	1332	45.9 (44.1-47.7)	261	17.4 (15.5-19.4)	1242	82.6 (80.6-84.5)
Manitoba	805	60.0 (57.3-62.6)	537	40.0 (37.4-42.7)	129	19.8 (16.9-23.0)	524	80.2 (77.0-83.1)
Alberta	842	64.3 (61.6-66.8)	468	35.7 (33.2-38.4)	146	20.3 (17.5-23.4)	574	79.7 (76.6-82.5)
British Columbia	1491	51.8 (49.9-53.6)	1389	48.2 (46.4-50.1)	237	17.1 (15.2-19.2)	1150	82.9 (80.8-84.8)
**Urbanicity of Residence**								
Urban	6736	54.2 (53.3-55.0)	5699	45.8 (45.0-46.7)	1073	17.4 (16.4-18.3)	5104	82.6 (81.7-83.6)
Rural	439	47.5 (44.3-50.7)	485	52.5 (49.3-55.7)	84	15.7 (12.8-19.0)	452	84.3 (81.0-87.2)
Missing	5	71.4 (32.7-92.8)	2	28.6 (7.2-67.3)	0	0.0 (N/A)	01	100 (N/A)
**Chronic medical condition status (self-reported)**								
None reported	1252	43.1 (41.3-44.9)	1652	56.9 (55.1-58.7)	342	6.1 (5.5-6.8)	5260	93.9 (93.2-94.5)
At least one reported	5795	56.7 (55.8-57.7)	4417	43.3 (42.3-44.2)	1157	17.2 (16.3-18.2)	5557	82.8 (81.8-83.7)
Missing	133	53.2 (47.0-59.3)	117	46.8 (40.7-53.0)	27	13.3 (9.3-18.7)	176	86.7 (81.3-90.7)
**Contact with family doctor in previous 12 months**								
No	259	36.6 (33.2-40.3)	448	63.4 (59.7-66.8)	40	7.2 (5.4-9.7)	512	92.8 (90.3-94.6)
Yes	6916	54.7 (53.8-55.5)	5734	45.3 (44.5-46.2)	1116	18.1 (17.2-19.1)	5042	81.9 (80.9-82.8)
Missing	5	55.6 (25.1-82.3)	4	44.4 (17.7-74.9)	01	25 (3.3-76.2)	03	75 (23.8-96.7)

Characteristic	Self-reported pneumococcal vaccination in lifetime							
	Individuals aged 65 and older (N=13,366)				Individuals aged < 65 with at least one CMC (N=6,714)			
	Vaccinated		Unvaccinated		Vaccinated		Unvaccinated	
	N	% (95% CI)	N	% (95% CI)	N	% (95% CI)	N	% (95% CI)
**Receipt of influenza vaccine in previous 12 months (self-reported)**								
No	842	21.7 (20.4-23.0)	3043	78.3 (77.0-79.6)	264	7.7 (6.9-8.7)	3158	92.3 (91.3-93.1)
Yes	6333	66.9 (65.9-67.8)	3133	33.1 (32.2-34.1)	893	27.1 (25.6-28.7)	2398	72.9 (71.3-74.4)
Missing	5	33.3 (14.6-59.4)	10	66.7 (40.6-85.4)	0	0.0 (N/A)	01	100 (N/A)
