# Supplementary material for: Changes in pneumococcal vaccine coverage in the Canadian Longitudinal Study on Aging (CLSA): An analysis based on the 2018–2021 follow-up 2 survey
Source: PLoS One. 2026 Jan 23;21(1):e0338213. doi: 10.1371/journal.pone.0338213 (PMC12829781; doi:10.1371/journal.pone.0338213)
Supplement: S3 Table — (PDF) [file pone.0338213.s003.pdf]

S3 Table. Distribution of chronic medical conditions (CMC) among individuals eligible for pneumococcal vaccination in comprehensive cohort, by self-reported pneumococcal vaccination status (vaccinated or unvaccinated during lifetime) for the period of FUP1 (2015-2018). Counts, percentages, and 95% confidence intervals within variable strata are shown for two subgroups of interest: 1) individuals aged 65 and older (n = 13,366), and 2) individuals aged 47-64 with at least one chronic medical condition (CMC) among those listed in the table (cardiovascular disease, chronic lung disease, cerebrovascular disease, chronic kidney disease, diabetes mellitus, cancer, chronic neurologic condition) (n = 6,714).

Characteristic	Self-reported pneumococcal vaccination in lifetime at FUP1							
	Individuals aged 65 and older (n=13,366)				Individuals aged < 65 with at least one CMC (n= 6,714)			
	Vaccinated		Unvaccinated		Vaccinated		Unvaccinated	
	N	% (95% CI)	N	% (95% CI)	N	% (95% CI)	N	% (95% CI)
**Overall**	7180	53.7 (52.9-54.6)	6186	46.3 (45.4-47.1)	1157	17.2 (16.3-18.2)	5557	82.8 (81.8-83.7)
**Chronic Medical Condition (self-reported)**								
None reported	1252	43.1 (41.3-44.9)	1652	56.9 (55.1-58.7)	342	6.1 (5.5-6.8)	5260	93.9 (93.2-94.5)
At least one reported	5795	56.7 (55.8-57.7)	4417	43.3 (42.3-44.2)	1157	17.2 (16.3-18.2)	5557	82.8 (81.8-83.7)
Missing	133	53.2 (47.0-59.3)	117	46.8 (40.7-53.0)	27	13.3 (9.3-18.7)	176	86.7 (81.3-90.7)
**Cardiovascular disease**								
No	2697	48.0 (46.7-49.3)	2917	52.0 (50.7-53.3)	470	16.2 (14.9-17.5)	2438	83.8 (82.5-85.1)
Yes	4360	58.0 (56.8-59.1)	3162	42.0 (40.9-43.2)	687	18.1 (16.9-19.3)	3119	81.9 (80.7-83.1)
Missing	123	53.5 (47.0-59.8)	107	46.5 (40.2-53.0)	0	0.0 (N/A)	0	0.0 (N/A)
**Chronic lung disease**								
No	5509	51.2 (50.3-52.1)	5251	48.8 (47.9-49.7)	626	14.1 (13.1-15.1)	3828	85.9 (84.9-86.9)
Yes	1540	65.3 (63.3-67.3)	819	34.7 (32.8-36.7)	527	23.4 (21.7-25.2)	1728	76.6 (74.8-78.3)
Missing	131	53.0 (46.8-59.2)	116	47.0 (40.8-53.2)	4	80.0 (16.3-18.2)	1	20.0 (2.7-69.1)
**Cerebrovascular disease**								
No	6414	53.2 (52.3-54.1)	5639	46.8 (45.9-47.7)	1084	16.9 (16.0-17.8)	5338	83.1 (82.2-84.0)
Yes	624	59.5 (56.5-62.5)	424	40.5 (37.5-43.5)	72	25.2 (20.5-30.5)	214	74.8 (69.5-79.5)
Missing	142	53.6 (47.6-59.5)	123	46.4 (40.5-52.4)	1	16.7 (2.3-18.2)	5	83.3 (36.9-97.7)
**Chronic kidney disease**								
No	6667	53.3 (52.4-54.1)	5847	46.7 (45.9-47.6)	1096	17.0 (16.1-18.0)	5334	83.0 (82.0-83.9)
Yes	367	62.6 (58.6-66.5)	219	37.4 (33.5-41.4)	60	21.6 (17.1-26.8)	218	78.4 (73.2-82.9)
Missing	146	54.9 (48.9-60.8)	120	45.1 (39.2-51.1)	1	16.7 (2.3-63.1)	5	83.3 (36.9-97.7)

Characteristic	Self-reported pneumococcal vaccination in lifetime at FUP1							
	Individuals aged 65 and older (n=13,366)				Individuals aged < 65 with at least one CMC (n= 6,714)			
	Vaccinated		Unvaccinated		Vaccinated		Unvaccinated	
	N	% (95% CI)	N	% (95% CI)	N	% (95% CI)	N	% (95% CI)
**Diabetes mellitus**								
No	5307	52.6 (51.6-53.6)	4779	47.4 (46.4-48.4)	680	14.5 (13.5-15.6)	4005	85.5 (84.4-86.5)
Yes	1731	57.4 (55.6-59.1)	1287	42.6 (40.9-44.4)	475	23.5 (21.7-25.4)	1548	76.5 (74.6-78.3)
Missing	142	54.2 (48.1-60.1)	120	45.8 (39.9-51.9)	2	33.3 (8.4-73.2)	4	66.7 (26.8-91.6)
**Cancer**								
No	5037	52.0 (51.0-53.0)	4652	48.0 (47.0-49.0)	903	16.8 (15.8-17.8)	4479	83.2 (82.2-84.2)
Yes	2013	58.7 (57.1-60.4)	1415	41.3 (39.6-42.9)	252	19.0 (17.0-21.2)	1076	81.0 (78.8-83.0)
Missing	130	52.2 (46.0-58.6)	119	47.8 (41.7-54.0)	2	50.0 (12.3-87.7)	2	50.0 (12.3-87.7)
**Chronic neurologic condition**								
No	6905	53.6 (52.7-54.4)	5983	46.4 (45.6-47.3)	1123	17.1 (16.2-18.0)	5444	82.9 (82.0-83.8)
Yes	152	61.3 (55.1-67.2)	96	38.7 (32.8-44.9)	34	23.1 (17.0-30.6)	113	76.9 (69.4-83.0)
Missing	123	53.5 (47.0-59.8)	107	46.5 (40.2-53.0)	0	0.0 (N/A)	0	0.0 (N/A)
