# Supplementary material for: Changes in pneumococcal vaccine coverage in the Canadian Longitudinal Study on Aging (CLSA): An analysis based on the 2018–2021 follow-up 2 survey
Source: PLoS One. 2026 Jan 23;21(1):e0338213. doi: 10.1371/journal.pone.0338213 (PMC12829781; doi:10.1371/journal.pone.0338213)
Supplement: S4 Table — (PDF) [file pone.0338213.s004.pdf]

**S4 Table. Distribution of chronic medical conditions (CMC) among individuals eligible for pneumococcal vaccination, by self-reported pneumococcal vaccination status (vaccinated or unvaccinated during lifetime) for the period of FUP2 (2018-2021).** Counts, percentages, and 95% confidence intervals within variable strata are shown for two subgroups of interest: 1) individuals aged 65 and older (n = 10,530), and 2) individuals aged 49-64 with at least one chronic medical condition (CMC) among those listed in the table (cardiovascular disease, chronic lung disease, cerebrovascular disease, chronic kidney disease, diabetes mellitus, cancer, chronic neurologic condition) (n = 4,055).

| Characteristic | Self-reported pneumococcal vaccination in lifetime FUP2 | | | | | | | |
|---|---|---|---|---|---|---|---|---|
| | Individuals aged 65 and older (n=10,530) | | | | Individuals aged < 65 with at least one CMC (n=4,055) | | | |
| | Vaccinated | | Unvaccinated | | Vaccinated | | Unvaccinated | |
| | N | % (95% CI) | N | % (95% CI) | N | % (95% CI) | N | % (95% CI) |
| **Overall** | 5976 | 56.8 (55.8-57.7) | 4554 | 43.2 (42.3-44.2) | 781 | 19.3 (18.1-20.5) | 3274 | 80.7 (79.5-81.9) |
| **Chronic Medical Condition (self-reported)** | | | | | | | | |
| None reported | 1107 | 46.5 (44.5-48.5) | 1272 | 53.5 (51.5-55.5) | 270 | 7.9 (7.1-8.9) | 3137 | 92.1 (91.1-92.9) |
| At least one reported | 4830 | 59.7 (58.7-60.8) | 3254 | 40.3 (39.2-41.3) | 781 | 19.3 (18.1-20.5) | 3274 | 80.7 (79.5-81.9) |
| Missing | 39 | 58.2 (46.2-69.4) | 28 | 41.8 (30.6-53.8) | 6 | 10.2 (4.6-20.8) | 53 | 89.8 (79.2-95.4) |
| **Cardiovascular disease** | | | | | | | | |
| No | 2397 | 51.8 (50.3-53.2) | 2234 | 48.2 (46.8-49.7) | 331 | 18.8 (17.0-20.7) | 1431 | 81.2 (79.3-83.0) |
| Yes | 3540 | 60.6 (59.4-61.9) | 2299 | 39.4 (38.1-40.6) | 450 | 19.7 (18.1-21.3) | 1839 | 80.3 (78.7-81.9) |
| Missing | 39 | 65.0 (52.2-75.9) | 21 | 35.0 (24.1-47.8) | 0 | 0.0 (N/A) | 4 | 100 (N/A) |
| **Chronic lung disease** | | | | | | | | |
| No | 4572 | 53.8 (52.8-54.9) | 3924 | 46.2 (45.1-47.2) | 420 | 15.8 (14.5-17.2) | 2237 | 84.2 (82.8-85.5) |
| Yes | 1354 | 69.1 (67.0-71.1) | 605 | 30.9 (28.9-33.0) | 358 | 25. 8 (23.5-28.1) | 1031 | 74.2 (71.9-76.5) |
| Missing | 50 | 66.7 (55.3-76.4) | 25 | 33.3 (23.6-44.7) | 3 | 33.3 (11.1-66.7) | 6 | 66.7 (33.3-88.9) |
| **Cerebrovascular disease** | | | | | | | | |
| No | 5420 | 56.5 (55.5-57.5) | 4177 | 43.5 (42.5-44.5) | 740 | 19.1 (17.9-20.4) | 3136 | 80.9 (79.6-82.1) |
| Yes | 496 | 58.9 (55.5-62.2) | 346 | 41.1 (37.8-44.5) | 41 | 23.7 (17.9-30.6) | 132 | 76.3 (69.4-82.1) |
| Missing | 60 | 65.9 (55.6-74.9) | 31 | 34.1 (25.2-44.4) | 0 | 0.0 (N/A) | 6 | 100 (N/A) |
| **Chronic kidney disease** | | | | | | | | |
| No | 5603 | 56.3 (55.3-57.3) | 4351 | 43.7 (42.7-44.7) | 745 | 19.1 (17.9-20.3) | 3164 | 80.9 (79.7-82.1) |
| Yes | 311 | 64.4 (60.0-68.5) | 172 | 35.6 (31.5-40.0) | 34 | 25.6 (18.9-33.6) | 99 | 74.4 (66.4-81.1) |
| Missing | 62 | 66.7 (56.5-75.5) | 31 | 33.3 (24.5-43.5) | 2 | 15.4 (3.9-45.1) | 11 | 84.6 (54.9-96.1) |
| **Diabetes mellitus** | | | | | | | | |
| No | 4478 | 55.9 (54.8-56.9) | 3538 | 44.1 (43.1-45.2) | 471 | 17.1 (15.7-18.5) | 2291 | 82.9 (81.5-84.3) |
| Yes | 1450 | 59.7 (57.7-61.6) | 979 | 40.3 (38.4-42.3) | 307 | 24.0 (21.8-26.4) | 971 | 76.0 (73.6-78.2) |
| Missing | 48 | 56.5 (45.8-66.6) | 37 | 43.5 (33.4-54.2) | 3 | 20.0 (6.6-47.0) | 12 | 80.0 (53.0-93.4) |
| **Cancer** | | | | | | | | |

| Characteristic | Self-reported pneumococcal vaccination in lifetime FUP2 | | | | | | | |
|---|---|---|---|---|---|---|---|---|
| | Individuals aged 65 and older (n=10,530) | | | | Individuals aged < 65 with at least one CMC (n=4,055) | | | |
| | Vaccinated | | Unvaccinated | | Vaccinated | | Unvaccinated | |
| | N | % (95% CI) | N | % (95% CI) | N | % (95% CI) | N | % (95% CI) |
| No | 4267 | 54.7 (53.6-55.8) | 3530 | 45.3 (44.2-46.4) | 616 | 18.9 (17.6-20.3) | 2642 | 81.1 (79.7-82.4) |
| Yes | 1697 | 62.7 (60.9-64.5) | 1008 | 37.3 (35.5-39.1) | 165 | 20.8 (18.1-23.7) | 630 | 79.2 (76.3-81.9) |
| Missing | 12 | 42.9 (26.2-61.3) | 16 | 57.1 (38.7-73.8) | 0 | 0.0 (N/A) | 2 | 100 (N/A) |
| **Chronic neurologic condition** | | | | | | | | |
| No | 5740 | 56.7 (55.8-57.7) | 4354 | 43.3 (42.3-44.2) | 774 | 19.3 (18.1-20.5) | 3244 | 80.7 (79.5-81.9) |
| Yes | 79 | 56.1 (48.2-63.7) | 62 | 43.9 (36.3-51.8) | 7 | 21.2 (10.5-38.3) | 26 | 78.8 (61.7-89.5) |
| Missing | 157 | 65.0 (52.2-75.9) | 138 | 35.0 (24.1-47.8) | 0 | 0.0 (N/A) | 4 | 100 (N/A) |
