# Supplementary material for: Changes in pneumococcal vaccine coverage in the Canadian Longitudinal Study on Aging (CLSA): An analysis based on the 2018–2021 follow-up 2 survey
Source: PLoS One. 2026 Jan 23;21(1):e0338213. doi: 10.1371/journal.pone.0338213 (PMC12829781; doi:10.1371/journal.pone.0338213)
Supplement: S5 Table — (PDF) [file pone.0338213.s005.pdf]

S5 Table: Main socio-demographic characteristics of CLSA participants who were asked or not asked about pneumococcal vaccination at follow-up 2 (FUP2).

Characteristic	Asked about pneumococcal vaccination							
	Individuals aged 65 and older (n=15,474)				Individuals aged < 65 with at least one CMC (n= 5387)			
	Not asked		Asked		Not asked		Asked	
	N	% (95% CI)	N	% (95% CI)	N	% (95% CI)	N	% (95% CI)
**Overall**	4438	28.7 (28.0-29.4)	11036	71.3 (70.6-72.0)	1140	21.2 (20.1-22.3)	4247	78.8 (77.7-79.9)
**Sex at birth**								
Female	2407	54.2 (52.8-55.7)	5409	49.0 (48.1-49.9)	654	57.4 (54.5-60.2)	2081	49.0 (47.5-50.5)
Male	2031	45.8 (44.3-47.2)	5627	51.0 (50.1-51.9)	486	42.6 (39.8-45.5)	2166	51.0 (49.5-52.5)
**Age group**								
<55	N/A	N/A	N/A	N/A	160	14.0 (12.1-16.2)	644	15.2 (14.1-16.3)
55-64	N/A	N/A	N/A	N/A	980	86.0 (83.8-87.9)	3603	84.8 (83.7-85.9)
65-74	2408	60.9 (59.4-62.4)	6086	55.1 (54.2-56.1)	N/A	N/A	N/A	N/A
75-84	1267	32.1 (30.6-33.5)	3646	33.0 (32.2-33.9)	N/A	N/A	N/A	N/A
85+	278	7.0 (6.3-7.9)	1304	11.8 (11.2-12.4)	N/A	N/A	N/A	N/A
**Racialized**								
No	4262	96.0 (95.4-96.6)	10542	95.5 (95.1-95.9)	1074	94.2 (92.7-95.4)	3945	92.9 (92.1-93.6)
Yes	172	3.9 (3.3-4.5)	481	4.4 (4.0-4.8)	66	5.8 (4.6-7.3)	300	7.1 (6.3-7.9)
missing	4	0.1 (0.0-0.2)	13	0.1 (0.1-0.2)	0	0.0 (N/A)	2	0.0 (0.0-0.2)
**Highest education level**								
Less than second. school educ.	273	6.2 (5.5-6.9)	672	6.1 (5.7-6.6)	31	2.7 (1.9-3.8)	104	2.4 (2.0-3.0)
Second. school grad., no post-second. school educ.	484	10.9 (10.0-11.9)	1051	9.5 (9.0-10.1)	118	10.4 (8.7-12.3)	304	7.2 (6.4-8.0)
Some post-second. educ.	308	6.9 (6.2-7.7)	910	8.2 (7.7-8.8)	72	6.3 (5.0-7.9)	309	7.3 (6.5-8.1)
Post-second. degree/diploma	3367	75.9 (74.6-77.1)	8376	75.9 (75.1-76.7)	919	80.6 (78.2-82.8)	3529	83.1 (81.9-84.2)
missing	6	0.1 (0.1-0.3)	27	0.2 (0.2-0.4)	0	0.0 (N/A)	1	0.0 (0.0-0.2)
**Annual household income (in Canadian dollars)**								
Less than $20,000	194	4.4 (3.8-5.0)	568	5.1 (4.7-5.6)	55	4.8 (3.7-6.2)	166	3.9 (3.4-4.5)
$20,000 to <$50,000	1257	28.3 (27.0-29.7)	2659	24.1 (23.3-24.9)	148	13.0 (11.2-15.1)	483	11.4 (10.5-12.4)

Characteristic	Asked about pneumococcal vaccination							
	Individuals aged 65 and older (n=15,474)				Individuals aged < 65 with at least one CMC (n= 5387)			
	Not asked		Asked		Not asked		Asked	
	N	% (95% CI)	N	% (95% CI)	N	% (95% CI)	N	% (95% CI)
$50,000 to <$100,000	1663	37.5 (36.1-38.9)	4059	36.8 (35.9-37.7)	370	32.5 (29.8-35.2)	1220	28.7 (27.4-30.1)
$100,000 to < $150,000	625	14.1 (13.1-15.1)	1719	15.6 (14.9-16.3)	263	23.1 (20.7-25.6)	995	23.4 (22.2-24.7)
$150,000 or higher	288	6.5 (5.8-7.3)	996	9.0 (8.5-9.6)	251	22.0 (19.7-24.5)	1161	27.3 (26.0-28.7)
missing	411	9.3 (8.4-10.1)	1035	9.4 (8.8-9.9)	53	4.6 (3.6-6.0)	222	5.2 (4.6-5.9)
**Province of residence**								
Newfoundland	370	8.3 (7.6-9.2)	679	6.2 (5.7-6.6)	125	11.0 (9.3-12.9)	289	6.8 (6.1-7.6)
Nova Scotia	511	11.5 (10.6-12.5)	1067	9.7 (9.1-10.2)	123	10.8 (9.1-12.7)	406	9.6 (8.7-10.5)
Quebec	801	18.0 (16.9-19.2)	2320	21.0 (20.3-21.8)	235	20.6 (18.4-23.1)	846	19.9 (18.7-21.1)
Ontario	887	20.0 (18.8-21.2)	2465	22.3 (21.6-23.1)	243	21.3 (19.0-23.8)	929	21.9 (20.7-23.1)
Manitoba	532	12.0 (11.1-13.0)	1032	9.4 (8.8-9.9)	111	9.7 (8.1-11.6)	399	9.4 (8.6-10.3)
Alberta	360	8.1 (7.3-9.0)	1128	10.2 (9.7-10.8)	110	9.6 (8.1-11.5)	427	10.1 (9.2-11.0)
British Columbia	977	22.0 (20.8-23.3)	2345	21.2 (20.5-22.0)	193	16.9 (14.9-19.2)	951	22.4 (21.2-23.7)
