# Supplementary material for: Changes in pneumococcal vaccine coverage in the Canadian Longitudinal Study on Aging (CLSA): An analysis based on the 2018–2021 follow-up 2 survey
Source: PLoS One. 2026 Jan 23;21(1):e0338213. doi: 10.1371/journal.pone.0338213 (PMC12829781; doi:10.1371/journal.pone.0338213)
Supplement: S6 Table — (PDF) [file pone.0338213.s006.pdf]

S6 Table. Results from primary versus sensitivity analysis for individuals ≥65 years (Objective 2). The sensitivity analysis was conducted by imputing missing income values at follow-up 2 (FUP2) with income values reported at FUP1.

Characteristic	Primary analysis (n=3134)			Sensitivity analysis with imputed income values (n= 3360)		
	aOR	95% CI		aOR	95% CI	
		Upper	Lower		Upper	Lower
**Sex at birth**						
Female (reference)	1.0			1.0		
Male	0.61	0.51	0.74	0.63	0.53	0.76
**Age group**						
65-74 (reference)	1.0			1.0		
75-84	0.70	0.58	0.85	0.70	0.59	0.84
85+	0.79	0.56	1.10	0.73	0.53	1.00
**Racialized**						
No (Reference)	1.0					
Yes	0.84	0.56	1.27	0.94	0.64	1.37
**Highest education level**						
Less than second. school educ. (reference)	1.0					
Second. school grad., no post-second. school educ.	1.20	0.77	1.86	1.11	0.73	1.71
Some post-second. educ.	0.90	0.56	1.46	0.85	0.54	1.35
Post-second. degree/diploma	1.06	0.72	1.55	1.06	0.73	1.53
**Annual household income (in Canadian dollars)**						
Less than $20,000 (reference)	1.0			1.0		
$20,000 to <$50,000	1.01	0.66	1.53	1.04	0.69	1.55
$50,000 to <$100,000	1.51	0.98	2.33	1.59	1.05	2.42
$100,000 to < $150,000	1.41	0.87	2.27	1.50	0.95	2.38
$150,000 or higher	1.39	0.83	2.34	1.47	0.89	2.43
**Marital/partner status**						

Characteristic	Primary analysis (n=3134)			Sensitivity analysis with imputed income values (n= 3360)		
	aOR	95% CI		aOR	95% CI	
		Upper	Lower		Upper	Lower
Single/never married/never lived with a partner (Reference)	1.0			1.0		
Married/Common-law	0.98	0.69	1.38	1.00	0.71	1.40
Widowed	0.74	0.49	1.09	0.83	0.57	1.21
Divorced/Separated	1.02	0.69	1.51	1.07	0.73	1.55
**Province of residence**						
Ontario (reference)	1.0			1.0		
Newfoundland	0.44	0.32	0.61	0.46	0.34	0.63
Nova Scotia	0.53	0.38	0.75	0.54	0.39	0.76
Quebec	1.60	1.20	2.11	1.58	1.21	2.08
Manitoba	1.03	0.70	1.50	1.08	0.77	1.52
Alberta	1.22	0.88	1.68	1.26	0.92	1.73
British Columbia	0.89	0.70	1.14	0.87	0.69	1.10
**Urbanicity of residence**						
Urban (Reference)	1.0			1.0		
Rural	0.88	0.64	1.21	0.87	0.64	1.18
**Chronic Medical Condition status (self-reported)**						
None reported (reference)	1.0			1.0		
At least one reported	1.17	0.94	1.44	1.15	0.94	1.41
**Change in chronic medical condition**						
No (Reference)	1.0			1.0		
Yes	1.41	1.01	1.96	1.35	0.98	1.85
**Receipt of influenza vaccination in the 12 months (self-reported at FUP2)**						
No (reference)	1.0			1.0		
Yes	6.70	5.40	8.32	6.31	5.13	7.75
**Contact with family doctor in previous 12 months**						

Characteristic	Primary analysis (n=3134)			Sensitivity analysis with imputed income values (n= 3360)		
	aOR	95% CI		aOR	95% CI	
		Upper	Lower		Upper	Lower
No (reference)	1.0			1.0		
Yes	1.94	1.35	2.79	2.01	1.41	2.85

aOR, Adjusted odds ratio; CI, Confidence interval
