# Supplementary figures and images for: Changes in pneumococcal vaccine coverage in the Canadian Longitudinal Study on Aging (CLSA): An analysis based on the 2018–2021 follow-up 2 survey
Source: PLoS One. 2026 Jan 23;21(1):e0338213. doi: 10.1371/journal.pone.0338213 (PMC12829781; doi:10.1371/journal.pone.0338213)

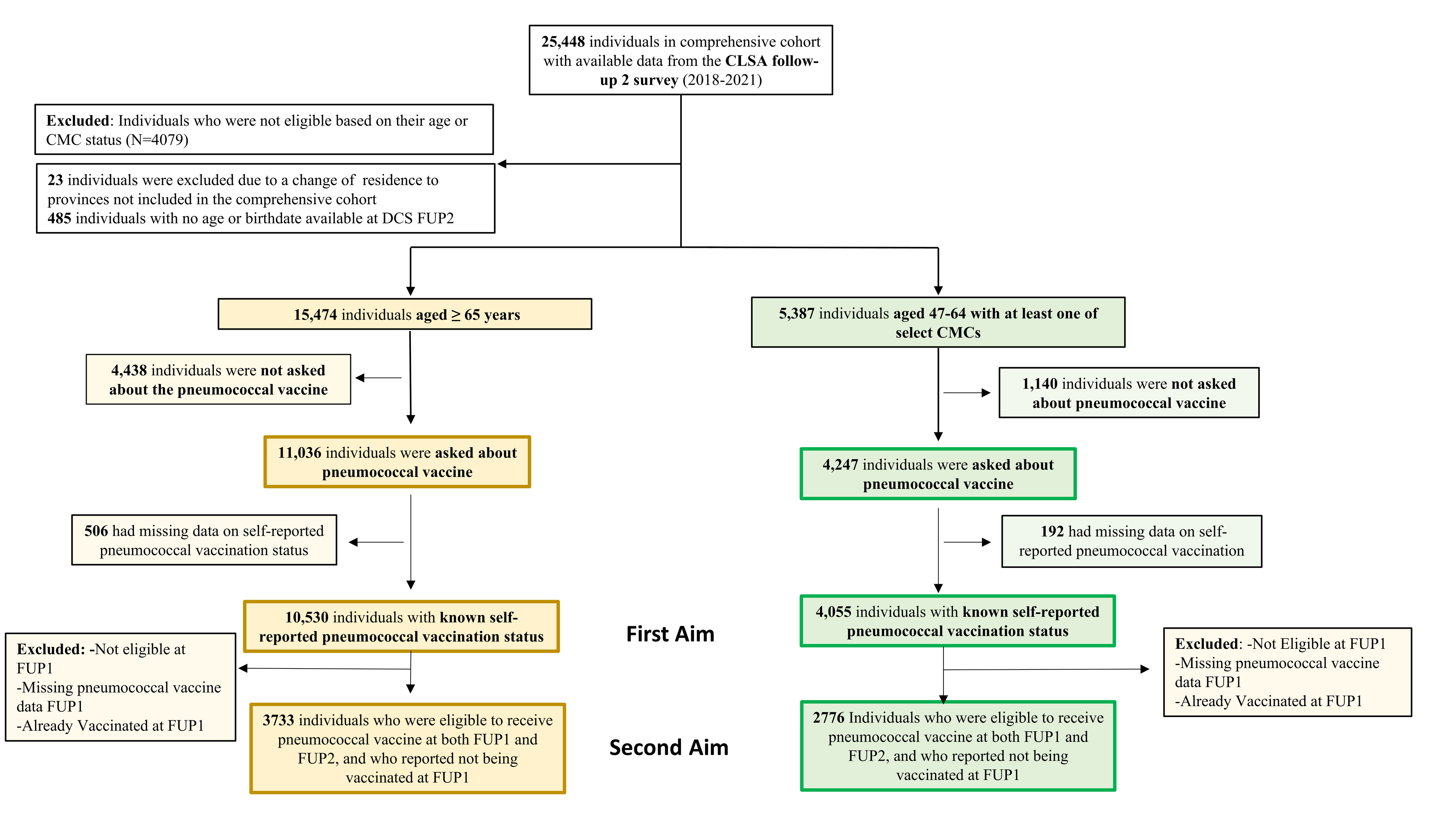

Supplement: S1 Fig — (TIF) [file pone.0338213.s009.tif]

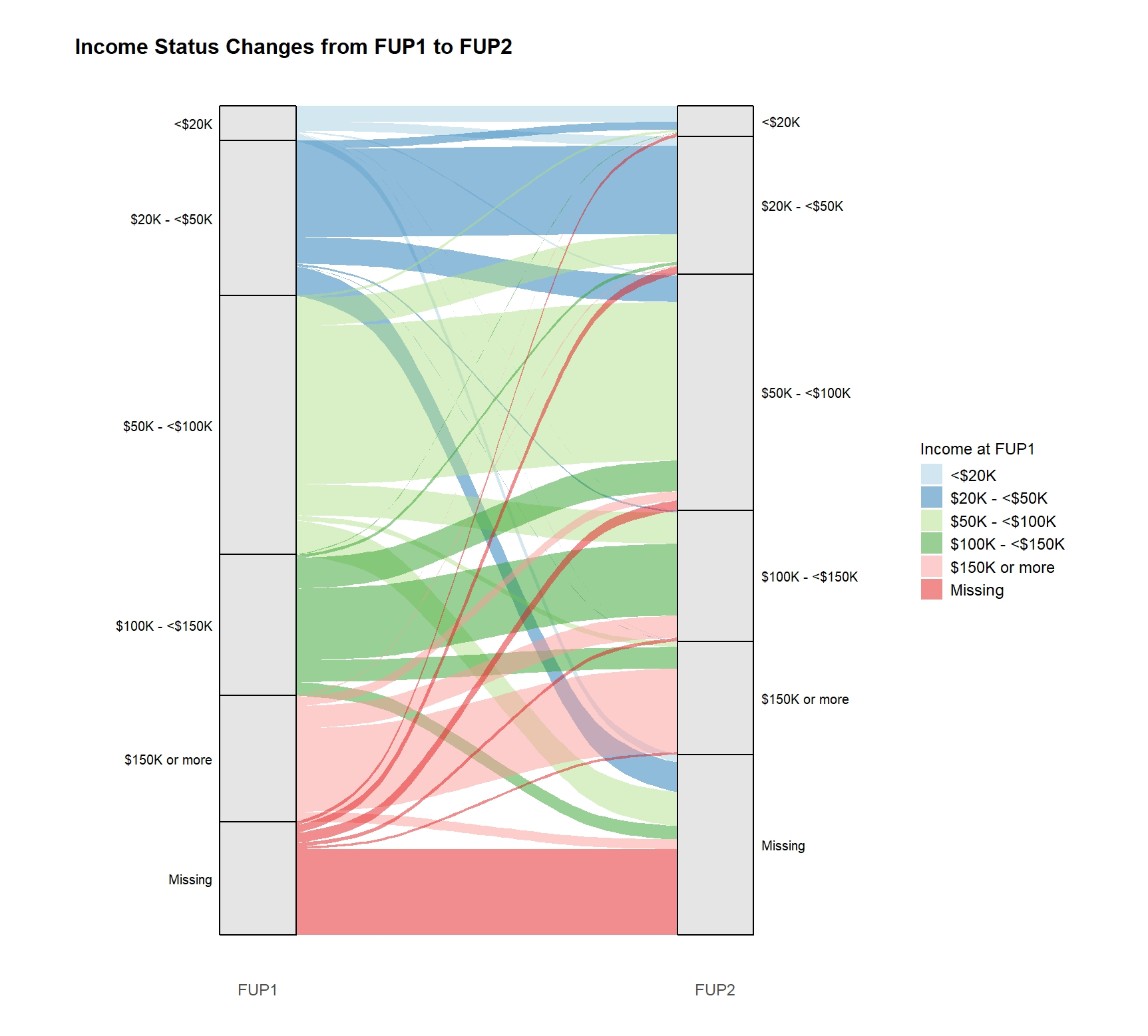

Supplement: S2 Fig — (TIF) [file pone.0338213.s010.tif]
