# Supplementary material for: Changes in pneumococcal vaccine coverage in the Canadian Longitudinal Study on Aging (CLSA): An analysis based on the 2018–2021 follow-up 2 survey
Source: PLoS One. 2026 Jan 23;21(1):e0338213. doi: 10.1371/journal.pone.0338213 (PMC12829781; doi:10.1371/journal.pone.0338213)
Supplement: Attachment — Submitted filename: CLSA FUP2 Pneumo vax_Sulis et al_Response to Reviewers.docx [file pone.0338213.s012.docx]

**PONE-D-25-27258 - [EMID:895b99d70c393abc]**

Changes in pneumococcal vaccine coverage in the Canadian Longitudinal Study on Aging (CLSA): An analysis based on the 2018-2021 follow-up 2 survey

**RESPONSE TO EDITOR’S AND REVIEWERS’ COMMENTS**

| **Reviewer** | **Comment** | **Response** |
| --- | --- | --- |
| Editorial office  Comment #1 | Please ensure that your manuscript meets PLOS ONE's style requirements, including those for file naming. The PLOS ONE style templates can be found at  <https://journals.plos.org/plosone/s/file?id=wjVg/PLOSOne_formatting_sample_main_body.pdf> and  [https://journals.plos.org/plosone/s/file?id=ba62/PLOSOne_formatting_sample_title_authors_affiliations.pdf](https://can01.safelinks.protection.outlook.com/?url=https%3A%2F%2Ftrack.editorialmanager.com%2FCL0%2Fhttps%3A%252F%252Fjournals.plos.org%252Fplosone%252Fs%252Ffile%253Fid%3Dba62%252FPLOSOne_formatting_sample_title_authors_affiliations.pdf%2F1%2F010f0197e2b315ec-78e5cba5-09ec-467f-8f7e-bd3029ffcfe5-000000%2Fru4lZaKbWH2r6ICw9tdoqtCpQEb4tFZOyDD9q367eIs%3D218&data=05%7C02%7Cgsulis%40uottawa.mail.onmicrosoft.com%7Ce319ec6da5f14d8981ca08ddbcfd6bcf%7Cd41fdab17e154cfdb5fa7200e54deb6b%7C1%7C0%7C638874519112966473%7CUnknown%7CTWFpbGZsb3d8eyJFbXB0eU1hcGkiOnRydWUsIlYiOiIwLjAuMDAwMCIsIlAiOiJXaW4zMiIsIkFOIjoiTWFpbCIsIldUIjoyfQ%3D%3D%7C0%7C%7C%7C&sdata=HrF7N2GDoKPsTvdWApbjGYYaWxw1mN%2F%2FkOICiqXhnV0%3D&reserved=0) | We have made the changes and ensured the manuscript and files follow PLOS ONE’s style and naming guidelines. |
| Editorial office  Comment #2 | For studies involving third-party data, we encourage authors to share any data specific to their analyses that they can legally distribute. PLOS recognizes, however, that authors may be using third-party data they do not have the rights to share. When third-party data cannot be publicly shared, authors must provide all information necessary for interested researchers to apply to gain access to the data. (<https://journals.plos.org/plosone/s/data-availability#loc-acceptable-data-access-restrictions>)  For any third-party data that the authors cannot legally distribute, they should include the following information in their Data Availability Statement upon submission:  1) A description of the data set and the third-party source  2) If applicable, verification of permission to use the data set  3) Confirmation of whether the authors received any special privileges in accessing the data that other researchers would not have  4) All necessary contact information others would need to apply to gain access to the data. | Please note that our “Data availability statement” already includes the required information:  “*Data are available from the Canadian Longitudinal Study on Aging (www.clsa-elcv.ca) for researchers who meet the criteria for access to de-identified CLSA data. For all approved users an interinstitutional CLSA Access Agreement is signed that prohibits the sharing of the CLSA data beyond the approved research team. Information about how to access the data via the CLSA data application process* can be found at https://www.clsa-elcv.ca/data-access/.”  Furthermore, all variables used for our analyses are listed and explained in S1 Table. |
| Editorial office  Comment #3 | Please include a caption for figure 1 and 2. | We have included the caption for figures 1 and 2 in the manuscript. |
| Editor  Comment #1 | We have received sufficient peer review reports on your manuscript, and we would like to share a summary of the feedback with you. Overall, the reviewers found your work timely and policy-relevant.  The reviewers have raised several important points that require your attention, particularly concerning the study design, analysis, and interpretation of findings. The most crucial points are highlighted below:  Major Points to address based on the reviews:  Study Design and Data Clarification Selection Bias and Missing Data Statistical Analysis and Survey Weights Model Validation and Goodness-of-Fit Impact of COVID-19 Pandemic Incorporation of Physician Contact  There are several other smaller comments but the major points are highlighted above.  We encourage you to carefully consider all feedback provided by the reviewers. Please revise your manuscript accordingly and provide a point-by-point response to each comment in your resubmission. | Thank you for the summary and guidance. We have carefully considered all reviewer feedback and revised the manuscript accordingly. A detailed, point-by-point response to each comment is provided below. |
| Reviewer #1  Comment 1 | The discussion briefly touches on provincial differences, but the paper could be strengthened by explaining how the findings might inform region-specific strategies, especially in provinces like Newfoundland, where vaccination rates were particularly low. | Thank you for this valuable suggestion. We have revised the Discussion section to briefly highlight how region-specific strategies could support increased pneumococcal vaccine uptake. In the revised text (lines 460-464), we note the importance of improving public awareness, supporting access through community-based care, and leveraging pharmacists to administer vaccines:  “*Together with local, community-based insights, these insights can inform region-specific strategies to boost vaccination rates. Approaches such as enhancing public awareness, improving access through community-based care, and utilizing pharmacists to administer the vaccine could help increase pneumococcal vaccine uptake among older adults [30–32]*.” |
| Reviewer #1  Comment 2 | Since some of the FUP2 data collection took place during the COVID-19 pandemic, it would be useful to add a short reflection on how this period may have affected vaccination behavior or access to care. This context could help clarify some of the observed trends. | Thank you for your comment. We agree that the COVID-19 pandemic likely influenced vaccination behaviors and access to healthcare services. In the Discussion section, we had already noted that the relatively stable pneumococcal vaccination rates observed during FUP2 – meaning rates that did not significantly change compared to FUP1 – may partly reflect pandemic-related service disruptions, reduced healthcare utilization, and shifts in healthcare-seeking behavior. We have now revised the sentence to clarify the interpretation and to note that further investigation is needed to determine the extent to which these factors may have impacted pneumococcal vaccination uptake (lines 442-449):  “*As part of the FUP2, data were collected during the early phases of the COVID-19 pandemic; the stagnant vaccination rates observed in this work could be potentially related to the disruption in vaccination services, the decreased utilization of healthcare services, and changes in vaccination behaviors related to the pandemic. However, further investigation is needed to determine if, and to what extent, disruption in vaccination services, decreased utilization of healthcare services, and changes in vaccination behaviors related to the COVID-19 pandemic may have influenced pneumococcal vaccination uptake among older adults during this period*.” |
| Reviewer #1  Comment 3 | It may be worth including a brief note to confirm that multicollinearity among predictors was checked and not found to be an issue. This would improve confidence in the stability of the regression estimates. | Thank you for your suggestion. Multicollinearity was assessed and found to be low. A brief note has been added to the Methods section (lines 267-268).  “*Multicollinearity among predictors was assessed using variance inflation factors (VIF), indicating low collinearity and stable regression estimates.”* |
| Reviewer #1  Comment 4 | While casewise deletion is reasonable given the low rate of missing data, a short explanation of why imputation was not used would demonstrate careful consideration of missingness, particularly in the case of income. | Thank you for the comment. We agree that income is a key variable, and we addressed this by imputing missing income data using values from FUP1. We then conducted a sensitivity analysis comparing results from models using complete case data without imputing income and models including imputed income. As reported in the Results section under "Sensitivity Analyses" (lines 410-415), the findings were consistent across the two model specifications, suggesting that missing income data did not significantly impact our results:  “*Given the amount of missing income data at FUP2, we investigated changes in reported income level between FUP1 and FUP2 and did not observe meaningful differences as illustrated in S2 Fig. In our sensitivity analyses for factors associated with pneumococcal vaccination for participants aged 49-64 with at least one CMC and participants aged 65 and older, we did not observe significant changes in adjusted model estimates obtained after imputing missing income data.(S6-S7 Tables).”* |
| Reviewer #2  Comment 1 | This manuscript used the newly released FUP2 CLSA data to study the uptake of pneumococcal vaccination and to identify factors associated with vaccination uptake compared with data from FUP1. The results are timely and policy relevant. Overall, this is a nicely written paper. The review wishes to see a bit more clarification on the study design and analysis, in particular with respect to selection bias, complete-case analysis, assessment of effect modification, and the choice of not using CLSA survey weights yet attempting to provide inference and conclusion aimed at national level estimates. I have enclosed detailed feedback below. | Thank you very much for your thorough review and feedback. Please find below our detailed responses to your comments. |
| Reviewer #2  Comment 2 | On longitudinal data. Can you clarify “non-overlapping follow-up surveys” post baseline? Does this mean that different individuals were surveyed at each time point? It’s a bit confusing here if non-overlapping is referring to non-overlapping participants or non-overlapping periods. The reviewer wishes to see clarification on whether the survey is done longitudinally, which features data collection on survey participants repeatedly. The reviewer does not think the data can be categorized as repeated cross-sectional data; it should just be termed as longitudinal data. | Thank you for this request for clarity. We agree that our original phrasing may have caused confusion. As noted in line 134, the CLSA is a longitudinal study in which the same individuals are followed over time and surveyed at approximately three-year intervals: baseline (2011-2015), follow-up 1 (2015-2018), and follow-up 2 (2018-2021). By "non-overlapping follow-up surveys," we were referring to the **distinct time periods** during which follow-up surveys were administered, not to different sets of participants – the wording has been modified for clarity (lines 127-131):  “*Longitudinal cohort participants enrolled in the CLSA during the baseline data collection period, which took place from 2011 to 2015. Subsequently, the same participants were invited to complete an additional round of data collection approximately every three years during CLSA follow-up data collection. The initial follow-up survey (FUP1) was conducted from 2015-2018. The next follow up survey (FUP2) was conducted from 2018-2021”*  We have also removed the term “repeated cross-sectional” from lines 143-145, as shown below:  *“We utilized the data collected from cohort participants during three distinct periods: the CLSA baseline study visit (conducted between 2011–2015), the CLSA FUP1 visit (conducted between 2015–2018), and the CLSA FUP2 visit (conducted between 2018-2021).”* |
| Reviewer #2  Comment 3 | The longitudinal data needs clarification, although age is not collected at FUP2 (exclude 485 participants) was age collected at baseline and FUP1? | Thank you for this comment. To clarify, age was collected at both baseline and subsequent follow-ups. For FUP2, we excluded 485 participants who were missing age data at that time point. These exclusions were based solely on missing age at FUP2, as this variable was required for the analyses. |
| Reviewer #2  Comment 4 | Again, clarification is needed as more than half of the FUP2 participants were excluded from the study cohort. | Thank you for pointing out the need for further clarity. The total number of participants in FUP2 was 25,448 (15,474 aged ≥65 years and 5,387 aged <65 years and with at least 1 CMC). However, due to an oversight in the administration of the survey, only 11,036 participants aged 65 years and older and 4,247 participants aged less than 65 years from FUP2 were asked about their pneumococcal vaccination status. Of these, 10,530 participants aged 65 and older [95.4% of that age group who were asked] and 4,055 participants under age 65 (95.4% of that age group who were asked) were included. Participants were excluded if they had missing age data or if they had missing self-reported pneumococcal vaccination status (n=506 for age 65 years and older; 4.5% of those asked and n=192 for those aged less than 65 years; 4.5% of those asked in that age group). The revised flow chart tracking participant eligibility and the inclusion and exclusion criteria is detailed in figure S1. |
| Reviewer #2  Comment 5 | On selection bias: Participants with missing data on self-reported pneumococcal vaccination status were excluded. Further inducing selection bias. Please justify the decision to exclude missing responses. Missing responses can be associated with vaccine uptake (that is informative missing), which may or may not be flagged by simply looking at demographic differences between the patient subgroups (as done in the sensitivity analysis). The reviewer suggests running a weighted logistic regression, weighted by the probability of missing self-reported vaccination status. This should be included as a sensitivity analysis. | Thank you for the comment. The proportion of missing responses was very small—only 4.5% in each age group. Specifically, among participants asked about pneumococcal vaccination status in FUP2, 95.4% of those aged ≥65 (10,530 of 11,036) and 95.4% of those aged <65 (4,055 of 4,247) responded. Given the low level of missingness, we believe the impact on the findings is likely minimal. |
| Reviewer #2  Comment 6 | Choice of statistical analysis for survey data: Please justify the rationale for not fitting the regression model properly, adjusting for the survey weights provided in the CLSA datasets to obtain national level estimates. | We agree that incorporating survey weights can be important for generating population-level estimates. However, at the time we conducted our analysis, the survey weights for the CLSA Follow-up 2 dataset had not yet been released. |
| Reviewer #2  Comment 7 | Please clarify what “logit transformation of proportions” means here. Are these univariate analyses by fitting multiple univariate logistic regressions for each of the factors by vaccination exposure? Again, see comments above, why not using survey weights here. If this was indeed the univariate analysis, please write the accurate statistical analysis term here. | These aOR results are not based on univariate analyses of multiple univariate logistic regression models. The aORs are estimated from models with multiple covariates as listed in the methods lines 259-265:  *“Specifically, our model for adults aged ≥65 years included age group, sex at birth, being racialized, highest education level, annual household income, marital/partner status, province of residence, urbanicity of residence, receipt of influenza vaccination in the 12 months prior to FUP2, reporting having been diagnosed with at least one CMC as of FUP2, and change in CMC status relative to FUP1. The model for adults < 65 years with CMCs included the same sociodemographic factors as outlined above along with receipt of influenza vaccination in the previous 12 months*. “  The “logit transformation” refers to the method used for estimating 95% CIs for the proportions only. |
| Reviewer #2  Comment 8 | Please justify the decision of not testing any interaction effect where the association between key sociodemographic factors can be moderated by history of chronic conditions as well as vaccination pattern and health care utilization in the previous 12 months.  Despite the large sample size (>10,000 total), fig1 and fig2 demonstrate wide confidence intervals for many covariates. Are there any model goodness-of-fit checks? Please provide a light analysis demonstrating the validity of the fitted logistic regression model. | We appreciate the thoughtful suggestions. Regarding interaction effects, our primary objective was to estimate main effects of key sociodemographic factors on pneumococcal vaccine uptake. While we acknowledge that interactions (e.g., between chronic conditions and healthcare utilization) could provide additional insight, testing such effects was beyond the scope of our current analysis and would require a more complex modeling strategy. In response to the reviewer’s second point, we have conducted goodness-of-fit checks to assess the validity of the fitted logistic regression model. Specifically, the Hosmer–Lemeshow test indicated acceptable model fit. Specifically, for the group age 65 years and above (P=0.19), for the age group less than 65 years (P=0.23). |
| Reviewer #2  Comment 9 | Consider changing the manuscript title to use of follow-up 2 to second wave. Follow-up 2 is a CLSA specific term where survey wave is generally accepted in wider context. | Thank you for the suggestion. We appreciate the point regarding terminology; however, the current phrasing comes from the CLSA documentation and terminology. |
| Reviewer #2  Comment 10 | In the introduction section, it would be helpful to introduce evidence on vaccine uptake globally. | Thank you for the suggestion. We have added the evidence on global vaccine uptake as well (see lines 75-77 and 88-92):  “*In response to the growing burden of pneumococcal disease, several countries across the globe have integrated adult pneumococcal vaccination into their National Immunization Programs as part of broader preventative health strategies [12]*.”  “*Globally, pneumococcal vaccine uptake among older adults varies widely but is generally low, with coverage ranging from approximately 18% in several European countries to around 59% in the United States and 37.8% in Japan, despite national immunization programs and public funding support [12,15]. In countries with publicly funded healthcare systems, such as the United Kingdom, coverage reaches 71.5%*.” |
| Reviewer #3  Comment 1 | On page 2, the manuscript asks, “Did you receive funding for this work?” but this question is left unanswered. However, funding is mentioned later on page 28. Please ensure that the funding information is clearly and consistently reported. | Thank you for pointing this out. All required statements were provided to the journal as requested. |
| Reviewer #3  Comment 2 | In the Introduction, when mentioning the second aim to identify factors associated with being newly vaccinated, I recommend specifying that these factors refer to sociodemographic variables to better align with the analysis, findings, and discussion. | Thank you for the suggestion. However, our analysis includes a comprehensive set of covariates known to influence vaccine uptake, primarily sociodemographic factors alongside clinical and healthcare utilization-related variables such as chronic medical conditions and prior influenza vaccination. We have modified the wording of the aim for clarity (lines 112-117).  “*Our second objective was to identify sociodemographic, clinical, and healthcare utilization factors associated with being newly vaccinated, specifically among those participants who reported having been vaccinated when surveyed during the 2018-2021 FUP2 survey but had previously reported not having received any pneumococcal vaccine during the 2015-2018 FUP1 survey, despite being eligible at both time points.”* |
| Reviewer #3  Comment 3 | Regarding the use of CLSA data, please clarify if the researchers applied for access and agreed to abide by the CLSA’s Data and Sample Access Policy | We confirm that access to the CLSA data was obtained through the formal application process, and we have noted this in the Methods (lines 137-138): *“… [analysis of] de-identified CLSA data accessed via an approved CLSA data request application.*”  This is also noted in the Acknowledgements: “*This research has been conducted using the Comprehensive Baseline v7.0, Comprehensive Follow-up 1 v4.0, and Comprehensive Follow-up 2 v1.0 data under Application Number 2206026.*” |
| Reviewer #3  Comment 4 | Please specify which software was used for data analysis | In the Methods (lines 268-270), we noted that: “*All analyses were conducted using the survey data commands in Stata version 18.0 (Stata-Corp, College Station, TX, USA) [24].*” |
| Reviewer #3  Comment 5 | The manuscript states: “The proportion of CLSA participants who were asked questions about pneumococcal vaccination was lower at FUP2 compared to FUP1. This decrease was due to a temporary, unexplained omission of certain sections of the survey questionnaire between early 2018 and early 2019.” Please clarify what is meant by “unexplained omission” in this context. | Thank you for pointing this out. We agree that the original phrasing (“unexplained omission”) was unclear. We have revised the sentence to clarify that the decrease in the number of participants asked about pneumococcal vaccination at FUP2 was due to administrative decisions made during the implementation of the survey. Specifically, the Public Health and Behaviours (PHB) module, which includes questions on pneumococcal vaccination, was temporarily removed from the survey between early 2018 and early 2019 and later reintroduced. Participants who completed the FUP2 survey during this period were not asked about their pneumococcal vaccination status, resulting in missing data for that portion of the cohort. We have updated the manuscript accordingly to reflect this clarification (line 282-286):  “*The proportion of CLSA participants who were asked questions about pneumococcal vaccination was lower at FUP2 compared to FUP1. This decrease resulted from an administrative decision during the implementation of the FUP2 survey, which temporarily removed the Preventive Health Behaviours (PHB) module, including the pneumococcal vaccination questions from the survey between early 2018 and early 2019*.” |
| Reviewer #3  Comment 6 | The finding that males consistently report lower vaccination rates compared to females requires further explanation. Could this be due to social norms, personal beliefs, or other factors? A brief discussion is recommended. | We acknowledge that exploring the underlying reasons for sex-associated disparities in vaccine uptake is important and we highlighted past research that has observed similar patterns in lines 453-457 of the Discussion:  “*Our findings indicate sex-associated disparities in self-reported pneumococcal vaccination status, with males consistently reporting lower vaccination rates compared to females. This observation aligns with other studies showing that females have a higher propensity for vaccination and often act as vaccination advocates within their families and communities [25,27,28]*.”  However, given the data available in our study, we cannot speculate on the reasons or explain the differences observed with any degree of certainty. We have highlighted the need to explore explanations in future studies in the limitations section (lines 530-534):  “*Future research could also explore how provincial vaccination policies, including differences in funding and delivery mechanisms, interact with both system-level and patient-level barriers to influence vaccine uptake, as well as the social, behavioral, and structural factors that may contribute to sex-related differences in vaccination coverage.”* |
| Reviewer #3  Comment 7 | Interestingly, higher income within the 49–64 age group with CMCs was found to be associated with lower vaccination rates, challenging prior assumptions that affordability is a primary barrier. Please discuss possible reasons why higher-income individuals in this group might have lower vaccination uptake | We recognize that the association between higher income and lower vaccination rates in the 49–64 age group with chronic medical conditions is somewhat unexpected. While this finding challenges common assumptions, exploring the underlying reasons in detail is beyond the current study’s scope. Potential explanations could include differences in perceived risk, health beliefs, or health-seeking behaviours, which are added to the discussion (line 482-483). *“The potential reasons for this may stem from differences in risk perception, health-seeking behavior, and health beliefs”* |
| Reviewer #3  Comment 8 | Contrary to general trends, higher education was found to be linked to lower vaccination odds among those aged 49–65 with CMCs, diverging from existing research suggesting positive correlations between education and vaccination. Please provide a clearer explanation or discussion of why this unexpected finding might occur. | The findings related to education and vaccine uptake were presented as observed in our analysis. As noted in the manuscript, we did not explore the underlying mechanisms behind these associations, as this was beyond the scope of the current study. We have focused on reporting the patterns identified within our dataset. |
| Reviewer #4  Comment 1 | Abstract section should be improved by focusing the significant statistical results of the study. The introduction section can be improved to focus on specific area of the study and authors may add novelty statement. | Thank you for the thoughtful feedback. We have reviewed the abstract and introduction and believe they appropriately reflect the focus, key findings, and objectives of the study within the allowed space. While we appreciate the suggestion to add a novelty statement, we feel the current framing sufficiently conveys the study’s contribution and scope. |
| Reviewer #4  Comment 2 | Author are suggested to improve the methodology section. Since the FUP2 vaccine questions were only asked of participants in the Comprehensive cohort, the restriction may lead to selection bias. Author should elaborate on how representative this subset is compared to the full CLSA cohort and whether weighting was used to address this. | Thank you for this valuable comment. However, this restriction was methodologically necessary, as pneumococcal vaccination status at FUP2 was only collected for participants in the Comprehensive cohort. Including participants from the Tracking cohort would have resulted in substantial missing outcome data and undermined the validity of our analysis. To ensure internal consistency and reliability of the findings, we focused on the subset of participants with complete and comparable data.  Regarding using sample weights, the sample weights were available after the completion of the analysis. |
| Reviewer #4  Comment 3 | The total eligible sample changes across aims (e.g., 10,530 vs. 3,733 for ≥65). Authors are suggested to include a flow diagram /CONSORT-style figure to clearly depict inclusion/exclusion criteria. Authors are suggested to include the STROBE check list (as an Appendix) and verify if they are complying with all the items on the STROBE check list. | Thank you for these recommendations. Please note that S1 Figure provides the detailed participant flow chart and indicates inclusions and exclusions at all levels. The STROBE Checklist has been completed and is now provided as S8 Table. |
| Reviewer #4  Comment 4 | The regression models include many sociodemographic covariates. Authors are suggested to clarify whether multicollinearity diagnostics (e.g., VIF) were performed and if model assumptions (e.g., goodness-of-fit) were validated. | Thank you for your suggestion. Multicollinearity was assessed, indicating low collinearity, and a brief note has been added to the results section (lines 395-397). We have conducted goodness-of-fit checks to assess the validity of the fitted logistic regression model. Specifically, the Hosmer–Lemeshow test indicated acceptable model fit. |
| Reviewer #4  Comment 5 | Data collection for FUP2 occurred during the COVID-19 pandemic (Lines 412–415). This may have artificially lowered vaccination rates due to healthcare access disruptions. A stratified analysis (e.g., pre- vs. post-2020) would strengthen the findings and help isolate pandemic-related effects. | Thank you for your suggestion. To clarify, FUP2 data collection occurred between 2018 and 2021, meaning only a portion of participants completed the survey after the onset of the COVID-19 pandemic. Additionally, the pneumococcal vaccination question asked about receipt of the vaccine in the previous 12 months, so for many participants—even those surveyed in 2020 or 2021—their responses may reflect vaccination behavior that occurred prior to the pandemic. Taken together, these factors suggest that the potential impact of COVID-19-related healthcare disruptions on reported vaccination rates in our sample is likely minimal. Furthermore, stratifying the analysis by pre- and post-pandemic timing is not feasible given the limited number of post-pandemic respondents. |
| Reviewer #4  Comment 6 | Although physician contact in the previous 12 months is noted (e.g., Lines 209–210, Table 1), its role is not explicitly modeled as a predictor of new vaccination. Given the public health relevance, authors should consider incorporating this variable into the adjusted models. | Thank you for raising this point. We agree that contact with a family doctor in the previous 12 months is an important factor and we have now incorporated this variable in our adjusted models. |
| Reviewer #4  Comment 7 | Authors are suggested to improve the discussion by linking findings to provincial vaccination policies (e.g., funding, public health messaging) and differentiating between system-level vs. patient-level barriers. | We appreciate this suggestion to further contextualize our findings in relation to provincial vaccination policies and to distinguish between system- and patient-level barriers. While we agree that these factors are important and relevant, detailed policy-level comparisons were beyond the scope of our current analysis. However, we have acknowledged these broader contextual influences in the discussion and recognize them as important directions for future research (lines 530-534):  “*Future research could also explore how provincial vaccination policies, including differences in funding and delivery mechanisms, interact with both system-level and patient-level barriers to influence vaccine uptake, as well as the social, behavioral, and structural factors that may contribute to sex-related differences in vaccination coverage.”* |
| Reviewer # 4 Comment 8 | Authors should describe any limitations of their study. Furthermore, these limitations should be discussed. | We appreciate the reviewer’s suggestion. We have described the key limitations of our study in the discussion section, including restrictions related to the FUP2 sample, the potential for recall bias in self-reported vaccination status, and the extent of missing income data. We also noted how these limitations were addressed, where possible, through sensitivity analyses and comparison with previously published findings (line 509-521) |
| Reviewer #4  Comment 9 | The manuscript is generally well-written, however, authors are suggested to improve the English language. There are minor grammatical inconsistencies and typographical errors especially in the Discussion and Conclusion section. Provide abbreviations used. | We thank the reviewer for the feedback. We have carefully reviewed the manuscript to address minor grammatical inconsistencies and typographical errors, particularly in the Discussion and Conclusion sections. Additionally, all abbreviations have been defined upon first use in the text. |
